# Evaluation of Alpha-Lipoic Acid and B Vitamins on Quality of Life and Metabolic Parameters among Diabetic Patients with Symptomatic Distal Symmetric Polyneuropathy: A Randomized Controlled Trial

**DOI:** 10.2174/0115733998451787260317083008

**Published:** 2026-04-03

**Authors:** Nani Draman, Che Nur Ain Che Abdullah, Noraini Mohamad, Zainab Mat Yudin, Wan Muhamad Amir W. Ahmad, Ritzzaleena Rosli Mohd Rosli, Chee Yong Chuan

**Affiliations:** 1 Department of Family Medicine, School of Medical Sciences, Health Campus, Universiti Sains Malaysia, Kubang Kerian, Kelantan, Malaysia;; 2 Medical and Basic Dental Sciences Unit, School of Dental Sciences, Health Campus, Universiti Sains Malaysia, Kubang Kerian, Kelantan, Malaysia;; 3 Biostatistics Unit, School of Dental Sciences, Health Campus, Universiti Sains Malaysia, Kubang Kerian, Kelantan, Malaysia;; 4 Neurology Unit, Department of Internal Medicine, School of Medical Sciences, Health Campus, Universiti Sains Malaysia, Kubang Kerian, Kelantan, Malaysia

**Keywords:** Alpha lipoic acid, blood pressure, distal symmetric polyneuropathy, metabolic profile, quality of life, vitamin B

## Abstract

**Introduction:**

Distal symmetric polyneuropathy (DSPN) is a debilitating diabetic complication that impairs Quality of Life (QoL). This study evaluated the effects of Alpha Lipoic Acid (ALA) plus B vitamins on QoL and metabolic parameters in diabetic patients with symptomatic DSPN.

**Methods:**

In this randomized, double-blind, placebo-controlled trial, 80 patients were allocated to receive either oral ALA plus B vitamins, *i.e.*, Bionerv^®^ (*n* =40) (intervention group) or placebo *(n* = 40) for 12 weeks. QoL, Body Mass Index (BMI), and Blood Pressure (BP) were assessed at baseline, 6 weeks, and 12 weeks. QoL was measured using the Revised Diabetes Quality of Life Questionnaire (Rv-DQoL), where higher scores indicate poorer QoL. Metabolic parameters (glycated haemoglobin (HbA1c), fasting blood sugar (FBS), fasting lipid profile (FLP) were assessed at baseline and 12 weeks.

**Results:**

In the intervention group, the mean Rv-DQoL score decreased significantly in within- group analysis from 29.50 ± 5.25 at baseline to 24.37 ± 3.43 at week 12 [F(2,156)=55.16; *p*-value <0.001]. However, between-group differences were not significant (*p*-value =0.151). Blood pressure also declined significantly within the intervention group (*p*-value <0.05), but no significant differences were observed between the placebo and intervention groups (*p*-value >0.05). Metabolic parameters (HbA1c, FBS, FLP, BMI) showed no significant changes between intervention and control groups (*p*-value >0.05).

**Discussion:**

Twelve-week treatment with ALA plus B vitamins resulted in improvements in QoL and BP among DSPN patients. However, these changes did not reach statistical significance compared with placebo, and no alterations in metabolic parameters were observed. The short duration and small sample size may have limited the detectability of effects.

**Conclusion:**

Twelve weeks of treatment with ALA and B vitamins resulted in an improvement in QoL and BP among DSPN patients. However, these changes did not reach statistical significance compared with placebo, and no alterations in metabolic parameters were observed.

**Clinical Trial registration No:**

ClinicalTrials.gov (NCT06568185).

## 1 INTRODUCTION

Diabetic Peripheral Neuropathy (DPN) is one of the microvascular complications of diabetes, with a global prevalence ranging from 26% to 39% depending on diagnostic criteria and the population studied [1-4]. Distal symmetric polyneuropathy (DSPN) is the most common and diffuse form of DPN, typically presenting with poor balance, numbness, tingling, burning, and stabbing pain sensations [5]. DSPN can be diagnosed using several clinical scoring systems, including the Neurological Symptom Score (NSS) and the Neuropathy Disability Score (NDS) [6] or Michigan Neuropathy Screening Instrument (MNSI) [7].

DSPN contributes substantially to morbidity and disability, often leading to complications such as impaired balance, risk of falls, impaired mobility, foot ulcerations, and an increased risk of lower-limb amputation [8-13]. Beyond physical complications, DSPN-related chronic pain causes sleep disturbances, anxiety, and depression, further diminishing the overall quality of life (QoL) [14]. It has been shown that patients with advanced-stage diabetic neuropathy experience significantly lower QoL, more intense pain, and a higher prevalence of comorbid conditions compared to those in earlier stages of the disease [15].

Despite its substantial impact on morbidity and QoL, DSPN treatment remains inadequate and challenging. Current first-line treatment for DSPN focuses primarily on symptomatic treatment of neuropathic pain, employing medications such as pregabalin, gabapentin, duloxetine, and tricyclic antidepressants [16]. These medications have demonstrated greater efficacy than a placebo in reducing neuropathic pain. While effective, their long-term use is often limited by adverse effects, such as nausea, somnolence, dizziness, reduced appetite, and constipation [17]. Therefore, Alpha-Lipoic Acid (ALA) and benfotiamine (vitamin B1) have been proposed as a pathogenetically oriented pharmacotherapy, which has been shown to be effective in the treatment of DSPN, especially for patients who are refractory to or intolerant of first-line pharmacotherapies [16, 17].

ALA is a potent antioxidant essential in reducing oxidative stress, which is one of the primary mechanisms underlying DSPN [18, 19]. Its multifaceted antioxidant properties have the potential to slow the progression or even reverse the effects of peripheral diabetic neuropathy [20, 21]. Several randomized controlled trials have shown that ALA is effective in treating DSPN [22-25]. A systematic review and meta-analysis published in 2025 concluded that oral ALA at doses ranging from 600 to 1,800 mg may be beneficial in improving or alleviating symptoms associated with DPN [26].

ALA has also been linked to lower serum triglyceride levels, enhanced insulin sensitivity, and improved overall QoL [27]. Supporting this, a study conducted in Egypt found oral supplements with ALA significantly improved glycemic control (reduction in HbA1c level), reduced body mass index (BMI) and Low-Density Lipoprotein (LDL-C), and increased High-Density Lipoprotein (HDL-C) levels [25]. Similarly, Agathos *et al.* reported that a 40-day course of ALA significantly alleviated neuropathic symptoms and lowered triglyceride levels, contributing to improved QoL in patients with diabetic neuropathy. However, no significant changes were observed in body weight, blood pressure, fasting glucose, or other lipid parameters compared to baseline [28]. Given the inconsistent findings regarding ALA’s effects on metabolic parameters, further research is needed to clarify its efficacy in improving metabolic profiles in patients with DSPN.

Simultaneously, neurotropic B vitamins, particularly thiamine (B1), pyridoxine (B6), and cobalamin (B12), are crucial in maintaining nerve health. Vitamin B1 functions as a targeted antioxidant, vitamin B6 regulates nerve metabolism, and vitamin B12 preserves myelin sheaths. Its deficiencies may lead to irreversible nerve damage and chronic pain, contributing to the development of peripheral neuropathy [29]. A systematic review concluded that B vitamin supplementation could improve many symptoms and signs of DPN [30]. Clinical evidence supports their therapeutic abilities in the treatment of patients with DPN with vitamin B12 for twelve months, who have observed increased plasma B12 levels and improved all neurophysiological parameters, pain score, and QoL [31].

Extensive research supports the individual therapeutic roles of ALA and B vitamins in managing DSPN, with ALA primarily acting by reducing oxidative stress [18], while B vitamins directly support nerve regeneration [29]. Combining ALA with B vitamins presents a rational and potentially synergistic therapeutic approach in treating Type 2 Diabetes Mellitus (T2DM) patients with symptomatic DSPN. However, the effect of this combination on improving QoL and metabolic outcomes remains inadequately explored. Therefore, this study aimed to evaluate the effects of ALA and B vitamin combination therapy on patient QoL and metabolic outcomes in T2DM patients with symptomatic DSPN.

## 2 METHODS

### 2.1 Design

The study design was a 12-week, single-center, randomized, double-blind, placebo-controlled clinical trial. This study’s protocol was registered with ClinicalTrials.gov (Identifier NCT06568185). To ensure the study adhered to recommended standards, it was carried out, and its results were reported in line with the guidelines of the Consolidated Standards of Reporting Trials (CONSORT) statement [32].

### 2.2 Population and Sample

Participant recruitment was conducted from June to December 2024 at the outpatient and diabetic clinic of Universiti Sains Malaysia Specialist Hospital. Eligible individuals were adults aged 18 years or older with underlying T2DM who were diagnosed with DSPN based on the NSS and NDS criteria [6]. Exclusion criteria included documented cognitive impairment, peripheral vascular disease (evidenced by non-palpable foot pulses or intermittent claudication), previous lower limb amputation, liver enzyme elevations (aspartate aminotransferase (AST) or alanine aminotransferase (ALT) levels exceeding three times the upper normal limit), renal dysfunction (chronic kidney disease stages IV or V), and the use of medications that could confound study outcomes such as antidepressants, anticonvulsants, opioids, neuroleptics, antioxidants, and especially methylcobalamin, pyridoxine, or other B-vitamin formulations. Pregnant or lactating women and individuals with known allergies to B vitamins (B1, B6, B12) or ALA were also excluded.

The required sample size was determined using G*Power software version 3.1.9.7. Sample size estimates were calculated for each of the two parameters of the planned statistical analysis using a repeated-measures ANOVA, which evaluates between-group and within-group effects. The largest calculated sample size was 35 participants per group, derived from the between-group analysis, assuming an effect size of 0.28, 80% power, a 95% confidence level, and a Type I error rate of 0.05. The final sample size was increased to 40 participants per group to accommodate a projected 10% attrition rate, resulting in a total study population of 80 individuals.

### 2.3 Assessment of DSPN

At the screening visit, all participants were evaluated for DSPN using the NSS and NDS scores. NSS is based on four symptom components: type, location, timing, and relief by maneuver. Symptom type: burning, numbness, or tingling = 2 points; fatigue or cramping = 1 point; no symptoms = 0 points. Symptom location: symptoms in the feet = 2 points; calves = 1 point; other areas = 0 points. Symptom timing: worsening at night = 2 points; present both day and night = 1 point; only during the day = 0 points. An additional point is added if symptoms wake the patient from sleep. The fourth symptom component is relief by maneuver: improved by walking = 2 points; by standing = 1 point; relief with sitting or lying down = 0 points. The maximum possible score is 9, with severity categorized as mild (3-4), moderate (5-6), or severe (7-9) [6, 33].

The NDS is obtained through a neurological examination that evaluates sensory functions, including vibration, temperature, pinprick sensation, and the Achilles tendon reflex in both feet. Vibration sense is assessed using a 128 Hz tuning fork placed on the hallux (big toe), with scoring as follows: 0 for normal and 1 for reduced or absent on each side [34]. Temperature perception is evaluated with a cold tuning fork on the dorsum of the foot, following the same scoring method [35]. Pain sensation is evaluated with a pinprick test applied to the proximal end of the big toenail. For each foot, the ability to distinguish between sharp and blunt is assessed: score 0 if sharpness is present, and score 1 if sharpness is absent [34]. The Achilles tendon reflex is examined bilaterally using a tendon hammer. Scoring is as follows: 0 if normal, 1 if the reflex appears only with reinforcement, and 2 if the reflex is absent [6, 33]. The total NDS can range from 0 to 10, with severity graded as mild (3-5), moderate (6-8), and severe (9-10) [6].

A diagnosis of DSPN in this study was established by the presence of moderate symptoms (NSS > 5) and mild signs (NDS > 3) [6]. A study by Asad *et al.* evaluated the sensitivity, specificity, and diagnostic accuracy of the NSS and NDS, using nerve conduction studies as a gold standard. The results showed that the NSS had a sensitivity of 82.05% and a specificity of 66.67%, while the NDS had a sensitivity of 92.31% and a specificity of 47.62%. The overall diagnostic efficacy of both scores was reported as 77% [36]. All data collection and neurological evaluations were conducted by two researchers who had received training and standardization under the guidance of a neurologist, ensuring consistency in assessment methods.

### 2.4 Study Enrollment and Randomization

Patients diagnosed with T2DM were recruited individually during their scheduled visits to the outpatient and diabetes clinic. Interested individuals were provided with a comprehensive explanation of the study’s purpose, objectives, and procedures. They were invited to a designated research room for screening, including performing the NSS and NDS to establish the diagnosis of DSPN. Then, those who met all inclusion and exclusion criteria were provided with written informed consent in hard copy prior to enrollment. Participants were then randomly assigned in a 1:1 ratio to either the intervention or control groups. Mixed block randomization was employed to maintain group balance. The randomization was performed by independent statisticians using IBM SPSS software, ensuring unbiased allocation to the intervention or control group. The randomization sequences were concealed in opaque, sealed envelopes.

### 2.5 Study Medication

The intervention group received a fixed-dose combination of ALA and B vitamins (Bionerv^®^), with each tablet containing 300 mg ALA, 500 mcg vitamin B12, 8 mg vitamin B6, and 39 mg vitamin B1. Participants took two tablets once daily after breakfast for 12 weeks, providing a total daily dose of 600 mg ALA, 1000 mcg vitamin B12, 16 mg vitamin B6, and 78 mg vitamin B1. The control group received placebo tablets comprising croscarmellose sodium, microcrystalline cellulose, silicon dioxide, and magnesium stearate, formulated to match the excipients of the active treatment. Blinding was maintained by using placebo tablets identical to the active formulation in appearance, size, and texture. Both groups followed the same dosing schedule throughout the study.

This treatment period was determined based on previously established clinical evidence and the anticipated timelines for peripheral nerve recovery, safety assessments, and trial feasibility. A 12-week period was allowed to observe clinically relevant improvements, while ensuring participant compliance and operational feasibility [37, 38].

### 2.6 Data Collection and Follow-up Visit

Data were collected at three time points: baseline (prior to the initiation of study medication), week 6, and week 12. During the first visit, participants' sociodemographic characteristics (such as age, gender, race, educational level, occupation, monthly income, marital status), medical profile (namely diabetes duration and medication, retinopathy, presence of comorbidities such as hypertension, dyslipidemia and ischemic heart disease) and the Revised Version of the Diabetes Quality of Life questionnaire (Rv-DQoL) were assessed. Systolic and diastolic blood pressure (SBP, DBP, respectively) were measured in a seated position using a calibrated automated device. After an overnight fast, 6 mL of venous blood was drawn for FBS, HbA1c, and FLP, which consists of total cholesterol (TC), LDL-C, and triglycerides (TG). In addition, renal function tests (RFT), including serum creatinine, and liver function tests (LFT), measuring aspartate transaminase (AST) and alanine transaminase (ALT), were performed as part of the baseline assessment.

During the 6-week follow-up, Rv-DQoL, BP, weight, and BMI were reassessed. The final visit during the 12-week period included repeat measurements of Rv-DQoL, BP, weight, BMI, FBS, HbA1c, and FLP.

The participants’ QoL was assessed during these three visits using the Rv-DQoL instrument. The Rv-DQoL is an adaptation of the original developed by Jacobson *et al.* specifically designed to assess QoL in individuals with T2DM [39]. The DQoL encompasses three primary domains: (i) Diabetes Life Satisfaction (QoL Satisfy), consisting of 18 items; (ii) Disease Impact Scale (QoL Impact), comprising 27 items; and (iii) Disease-Related Worries Scale (QoL Worry), containing 14 items, along with a single general item evaluating overall self-perceived health status. All items in the QoL Satisfy domain are rated on a five-point Likert scale from 1 (very satisfied) to 5 (very dissatisfied). Items within the QoL Impact and QoL Worry domains are also rated on a five-point scale, ranging from 1 (never) to 5 (all the time). Scores for each domain are calculated by summing the item scores and dividing by the number of items, yielding an average score. Higher average mean scores indicate poorer QoL [39]. A Malay version of the instrument was developed in 2018, keeping the three main domains of ‘satisfaction’ (6 items), ‘impact’ (4 items), and ‘worry’ (3 items) with a similar score scale. Each domain’s redundant questions were removed, leaving the newly revised Rv-DQoL with a total of 13 questions. Cronbach’s α values for each domain ranged from 0.75 to 0.93, indicating good internal consistency, and it was validated for use among adult T2DM patients in Malaysia. It has a 5-point Likert scale ranging from 1 (very satisfied) to 5 (very dissatisfied) for the ‘satisfaction’ domain. For the ‘impact’ and ‘worry’ domain, it was rated as 1 (never), 2(sometimes), 3 (often), 4 (frequently) and 5 (always). A higher score indicates a lower QoL [40]. The use of the questionnaires was granted by the authors.

### 2.7 Study Outcomes

The primary outcome of the study is a change in Rv-DQoL, and secondary outcomes are changes in metabolic parameters (HbA1c, FBS, FLP (TC, LDL-C, TG), BMI, SBP, DBP) among participants with DSPN during the 12-week intervention period. This would provide a comprehensive evaluation of the intervention's therapeutic effects on patient-reported QoL and metabolic control.

### 2.8 Compliance and Safety Measures

Participants were provided with a daily checklist to document their intake of the assigned medication, ensuring adherence to the study medication. Participants were instructed to mark the checklist each day after consuming medication. Compliance was defined as the ingestion of at least 80% of the prescribed doses throughout the study. Participants who did not comply with the study protocol were withdrawn from the study and not substituted.

Safety was evaluated by continuous monitoring of adverse events (AEs) and Serious Adverse Events (SAEs) throughout the study. Participants were instructed to promptly report any adverse symptoms, which were documented using a standardized adverse event reporting form. Any event suspected of being related to the study intervention was reported to the clinic and evaluated by a qualified physician from the research team, who determined causality and provided appropriate clinical management when necessary. Participants who experienced Serious Adverse Events (SAEs) were withdrawn from the study and were not replaced.

### 2.9 Statistical Analysis

All statistical analyses were performed using IBM SPSS Statistics, version 29 (IBM Corp., Armonk, NY, USA) [41] and R (version 4.3.1; R Foundation for Statistical Computing, Vienna, Austria) [42]. Analyses adhered to the intention- to-treat (ITT) principle, including all randomized participants who received at least one dose of the study medication. To handle missing outcome data, a non-parametric random-forest-based multiple imputation was performed using the missRanger package in R, which applies chained random forests with predictive mean matching to preserve the original data structure and distribution [43]. Diagnostic plots comparing observed and imputed data confirmed that the imputation preserved the distributions of the underlying variables.

For categorical variables, differences between treatment groups were assessed using the chi-square test or Fisher’s exact test, as appropriate. Continuous variables were checked for normality using the Shapiro-Wilk test and summarized as mean and Standard Deviation (SD). Between-group comparisons at baseline were examined using independent t-tests.

Primary and secondary outcomes were analyzed using repeated-measures analysis of variance (RM-ANOVA) to evaluate both (i) within-group changes over time (time effect) and (ii) overall differences between treatment groups (group effect). The interaction between time and treatment (time * group effect) was also examined. Model results were presented as mean (SD) and 95% Confidence Intervals. When Mauchly’s test indicated sphericity violations, the Greenhouse-Geisser correction was applied. Statistical significance was set at *p*-value < 0.05 (two-tailed).

A per-protocol (PP) analysis was subsequently performed, including only participants who completed the intervention with ≥ 80% medication adherence and without major protocol deviations, to verify the robustness of ITT findings.

## 3 RESULT

Of the 82 individuals screened, 80 met the inclusion criteria and were randomized equally to the intervention (ALA + B-vitamins) and placebo groups (Fig. **1**). Two participants were excluded due to elevated serum creatinine levels. During follow-up, six participants (7.5%) discontinued the study, with two (5%) in the intervention arm and four (10%) in the placebo arm, primarily due to mild adverse effects. Following random-forest imputation of missing values for ITT analysis, distributional diagnostics confirmed close agreement between observed and imputed datasets, indicating that imputation did not materially distort variable distributions. Hence, the ITT dataset (*n* = 80) was used for the primary analysis, while the PP dataset (*n* = 74) was analyzed for sensitivity comparison.

Baseline sociodemographic and clinical characteristics were comparable between groups (Table **1**), with no statistically significant differences. Similarly, baseline Revised Diabetes Quality of Life (Rv-DQoL) scores and metabolic parameters did not differ significantly between the intervention and placebo groups (Table **2**), confirming randomization balance prior to intervention.

Table **3** presents a comparison of Rv-DQoL over a 12-week period. The time treatment interaction effect Rv-DQoL was not significant, indicating no differences between the intervention and placebo groups at each time point (*p*-value = 0.151). However, within-visit analysis (time effect) from baseline to 12 weeks in the intervention group revealed a significant effect over time [F-stat (df) = 55.16 (2, 156); *p*-value < 0.00^1^].

Table **4** summarizes changes in metabolic parameters over 12 weeks. The time treatment interaction effects for BMI, SBP, DBP, FBS, HbA1c, and FLP (TC, LDL-C, TG) were not significant, indicating no differences between the intervention and placebo groups at each time point (*p*-value > 0.05). However, within-visit analysis (time-effect) in the intervention group revealed significant reductions in SBP [F-stat (df) = 18.08 (2, 156); *p*-value < 0.00^1^] and DBP [F-stat (df) = 4.93 (2, 156); *p*-value = 0.00^8^].

### 3.1 Adverse Event

Reported side effects generally occur at low frequencies in both groups. During the second visit, nausea (15%) was the most common adverse effect in the intervention group, followed by itchiness (5%), dizziness (5%), and light-headedness (5%). A similar adverse event was reported in the placebo group, with nausea (5%), itchiness (2.5%), dizziness (2.5%), and light-headedness (2.5%). At the third visit, only two participants reported mild but tolerable nausea: one (2.5%) from the intervention group and one (2.5%) from the placebo group (Table **5**).

Two participants (5%) from the intervention group discontinued the study medication due to adverse effects, including nausea and itchiness. In the placebo group, four participants (10%) discontinued the study treatment. Of these, two participants discontinued taking medication due to medical conditions deemed unrelated to the study: one experienced femoral pain following a plating procedure, and another had an acute exacerbation of bronchial asthma. All adverse events were reviewed by the study investigator and were determined to have an “unlikely” causal relationship with the investigational product. The remaining two participants in the placebo group discontinued due to nausea. No serious adverse events were reported throughout the study duration.

## 4 DISCUSSION

DSPN remains a major chronic complication of T2DM that profoundly affects patients’ QoL and daily functioning [44]. This study found that the within-visit (time-effect) analysis demonstrated significant improvements in Rv-QoL scores in the intervention group after 12 weeks of treatment. However, the difference was not statistically significant compared to the placebo group. Our findings align with a study conducted in Greece, which examined the effect of ALA 600 mg daily on patients with painful diabetic neuropathy (*n*=72) over a 40-day period. The study reported significant QoL improvement in terms of work disability, social life disability, and family life disability [28]. Improvement in QoL was also observed in diabetic neuropathy patients receiving vitamin B12 supplementation for 12 months, as compared to the placebo group [31].

The lack of statistical significance in QoL between the intervention and placebo groups in this study may reflect the relatively short 12-week study duration, as previous studies suggest that more pronounced benefits are observed with longer treatment periods [45]. Moreover, the Rv-DQoL questionnaire used in this study emphasized domains related to satisfaction, the impact of diabetes mellitus, and worry domains, which primarily capture psychological aspects rather than physical functioning. Supporting this view, a study in Ukraine found that increased physical activity among patients with DPN significantly improves their QoL and represents a clinically effective approach because it reduces impaired coordination, builds confidence, and thus improves their emotional state and QoL in general [46]. Additionally, a smaller sample size in this study may contribute to a non-significant difference between the groups. Evidence suggests that a small sample size may not have sufficient power to detect clinically important differences in effect sizes [47].

For metabolic outcomes, no significant differences were observed between the intervention and placebo groups in BMI, SBP, DBP, FBS, HbA1c, and FLP (TC, LDL-C, TG) after a 12-week treatment period. Similar findings were reported in a study conducted in Greece that found no statistically significant difference in FBS, TC, LDL-C, HbA1c, BMI, and BP after 40 days on 600mg of ALA [28]. Another study in Korea also found there were no significant differences between the ALA and placebo groups in terms of BMI, FBS, HbA1c, TC, LDL-C, or TG after 12 weeks of treatment [37]. Similarly, a study by Didangelos *et al.* also found that there was no significant difference in SBP, DBP, TC, TG, HDL, LDL-C, and HbA1c after 12 months on superoxide dismutase, ALA, vitamin B12, and carnitine as compared to the placebo group [48].

Nevertheless, some studies have reported significant outcomes. A study by Siddique *et al.* observed a significant reduction in HbA1c after six months of supplementation with 600 mg of ALA, while Elbadawy *et al.* reported similar improvements in HbA1c, as well as reductions in BMI and LDL-C, following 12 weeks of treatment with 600mg of ALA [24, 25]. It was reported that ALA supplementation is associated with significantly reduced serum TG, improved insulin sensitivity, and enhanced QoL. These benefits are thought to arise from ALA’s anti-inflammatory, anti-hyperglycemic, and antioxidant properties, as well as its regulatory effects on endothelial function, insulin sensitivity, and lipid metabolism [27, 49].

In this study, the within-visit analysis (time-effect) shows a statistically significant reduction in both SBP and DBP from baseline to week 12 in the intervention group, suggesting that ALA combined with B vitamins may exert a beneficial effect on BP regulation over time. This aligns with experimental findings showing that ALA can attenuate renal and vascular endothelin-1 production and restore calcium levels, thereby lowering blood pressure in hypertensive animal models [19, 27, 50].

The mixed findings reported across studies may be explained by several factors. Firstly, variability in baseline characteristics such as glycemic control, lipid profiles, blood pressure, and BMI can strongly influence the metabolic response to supplementation. Secondly, differences in concurrent pharmacotherapies, particularly agents used to control blood glucose, lipids, and BP, may confound the observed effects of the ALA and B vitamins combination. Finally, our study did not account for dietary intake, physical activity, or adherence to lifestyle recommendations, all of which may introduce additional variability and influence outcomes.

## 5 STUDY LIMITATIONS

Our study has several limitations. First, according to the American Diabetes Association (ADA) statement in 2010, DSPN diagnosis required both abnormal Nerve Conduction Studies (NCS) and the presence of symptoms or signs of neuropathy [51]. However, in the 2017 ADA statement, it was stated that the diagnosis of DSPN is primarily clinical, based on typical symptoms and signs of symmetrical distal sensory loss, with electrophysiological testing reserved for atypical, uncertain cases or when another underlying cause is suspected [5, 52]. In the present study, NCS was not performed, and diagnosis of DSPN was established using validated clinical tools, namely the NSS and NDS scores, which have been widely applied to establish the diagnosis of DSPN in previous studies conducted in Brazil, Germany, the United Kingdom, and Malaysia [33, 53-55]. Future research should incorporate NCS results to strengthen the diagnostic accuracy of DSPN. Additionally, this study is a single-centre design, which may constrain the generalizability of the findings to broader populations. Moreover, the relatively short follow-up period restricts the evaluation of long-term efficacy outcomes. Also, the relatively small sample size in this study may have limited statistical power to detect smaller but clinically meaningful differences in QoL or metabolic parameters between groups. To enhance the robustness and external validity of future research, multi-centre studies with larger sample sizes, more diverse cohorts and extended follow-up durations are recommended.

## CONCLUSION

This study demonstrated that a 12-week treatment with ALA and B vitamins (intervention group) showed no significant differences in FBS, HbA1c, LDL-C, TG, SBP, DBP, and Rv-QoL compared to the placebo group. These findings do not support the hypothesis that a combination of ALA and B vitamins provides measurable benefits on glycemic control, lipid profiles, or QoL within the study population.

However, within-group analysis revealed significant improvements in Rv-DQoL and blood pressure among participants in the intervention group, suggesting the need for further exploration. The intervention group demonstrated favourable short-term tolerability over the 12-week period, with only mild, self-limiting adverse events reported and metabolic parameters remaining stable throughout the study.

These findings highlight the need for a larger sample size and longer-term studies with extended follow-up to validate the observed benefits and to explore potential metabolic advantages, thereby establishing the clinical relevance of this intervention.

## Figures and Tables

**Fig. (1):**
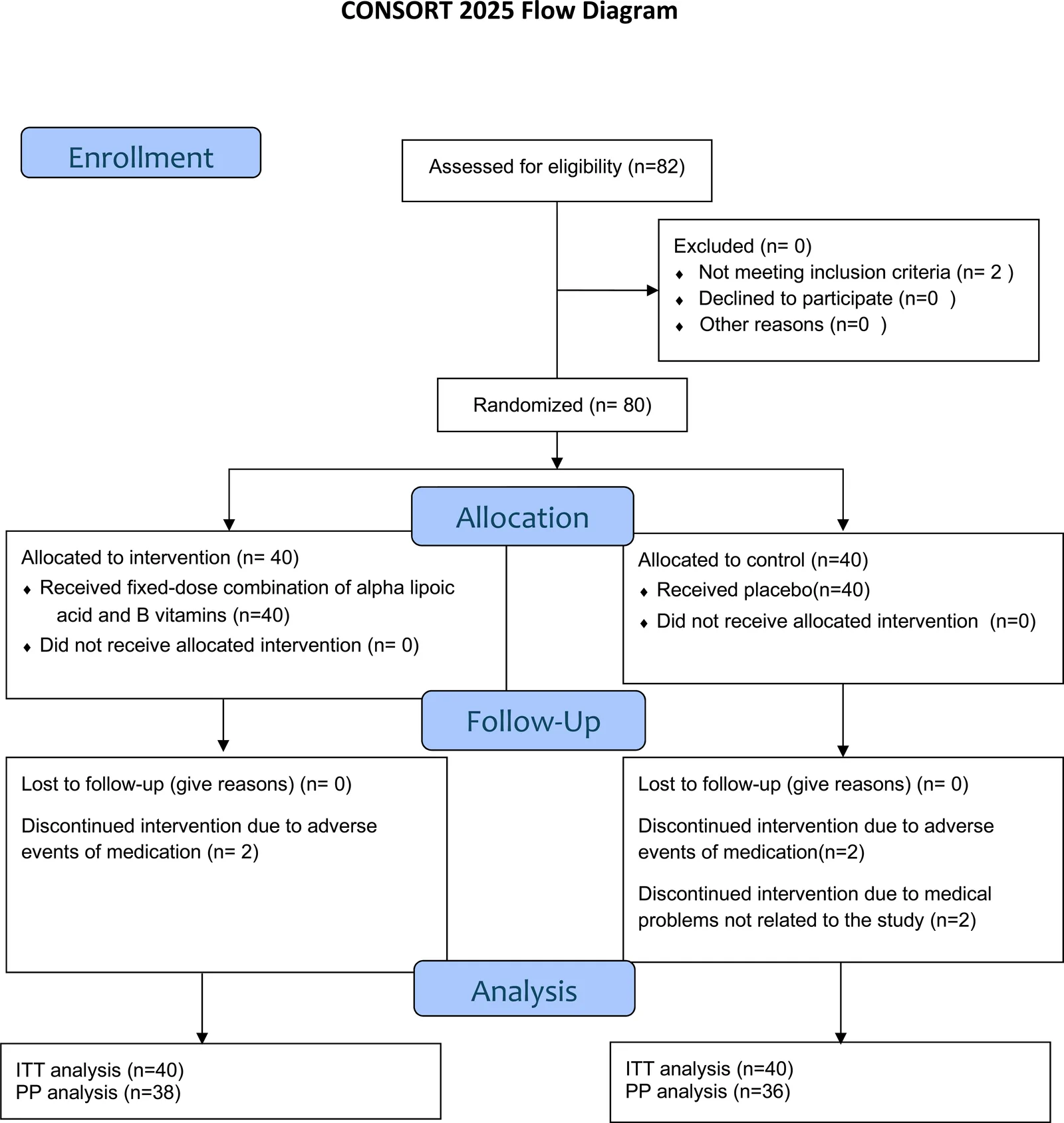
The subject distribution throughout the trial based on the CONSORT flow diagram [32].

**Table 1:** Baseline sociodemographic data and medical profile of the respondents (*n*=80).

Variables	Intervention (*n*=40) *n*(%)	Placebo (*n*=40) *n(*%)	Total (*n*=80) *n*(%)	*p*-value
**Age (year)**	65.40 (7.05)*	62.95(10.77)*	64.18 (9.13)*	0.233^a^
**Gender**				
Male	17 (43.6)	22 (56.4)	39 (48.8)	0.423^b^
Female	23 (56.1)	18 (43.9)	41 (51.2)	0.435^b^
**Race**				
Malay	36 (49.3)	37(50.7)	73 (91.2)	0.907^b^
Others	4 (57.1)	3 (42.9)	7 (8.8)	0.705^b^
**Education**				
No/ primary school	3 (42.9)	4 (57.1)	7 (8.8)	0.705^b^
Secondary school	29 (59.2)	20 (40.8)	49 (61.2)	0.199^b^
Tertiary level	8 (33.3)	16 (66.7)	24 (30.0)	0.102^b^
**Occupation**				
Employed	5 (38.5)	8 (61.5)	13 (16.2)	0.405^b^
Unemployed	35 (52.2)	32 (47.8)	67 (83.8)	0.714^b^
Monthly Income (MYR)	2424.48* (1890.75)	2935.75* (1743.25)	2680.11* (1825.17)	0.212^a^
**Marital status**				
Single/widow	7 (50.0)	7 (50.0)	14 (17.5)	1.000^b^
married	33 (50.0)	33 (50.0)	66 (82.5)	1.000^b^
Duration of DM (years)	16.18 (6.40)*	15.68 (7.98)*	15.93 (7.19)*	0.758^a^
**Medication**				
Metformin	30 (44.8)	37 (55.2)	67(83.8)	0.392^b^
Gliclazide	8 (38.1)	13 (61.9)	21(26.3)	0.275^b^
SGLT2 inhibitors	16 (57.1)	12 (42.9)	28(35.0)	0.450^b^
DPP-4 inhibitors	3 (60.0)	2 (40.0)	5 (6.3)	0.655^b^
Insulin	32 (53.3)	28(46.7)	60 (75.0)	0.606^b^
Hypertension	37 (51.4)	35 (48.6)	72 (90.0)	0.814^b^
Hyperlipidemia	39 (49.4)	40 (50.6)	79 (98.8)	0.910^b^
Retinopathy	14 (56.0)	11 (44.0)	25 (31.3)	0.549^b^
Ischemic heart disease	11 (45.8)	13 (54.2)	24 (30.0)	0.683^b^

*mean (SD) ^a^ Independent T-Test, ^b^ Chi-Square test.

**Table 2:** Baseline Rv-DQoL and clinical parameters of respondents (*n*=80).

**Variable**	**Mean (SD)**		**Mean Diff** **(95%CI)**	**t-stat (df)**	*p*-value*
	**Intervention** **(*n*=40)**	**Placebo** **(*n*=40)**			
Score Rv-DQoL	29.39 (5.35)	29.86 (5.84)	-0.47 (-3.06, 2.13)	-0.31(78)	0.759
Height (meters)	1.57 (0.08)	1.60 (0.08)	-0.031 (-0.07, 0.01)	-1.60 (78)	0.113
Weight (kg)	72.66 (12.26)	72.11 (18.32)	0.545 (-6.41, 7.50)	0.16(68)	0.876
BMI (kg/m^2^)	29.21 (3.37)	27.80 (6.04)	1.41 (-0.76, 3.59)	1.29 (61)	0.201
SBP (mmHg)	138.00 (18.00)	135.43 (20.40)	2.57 (-5.99, 11.14)	0.59 (78)	0.551
DBP (mmHg)	78.45 (11.12)	79.55 (11.45)	-1.10 (-6.13, 3.93)	-0.43 (78)	0.664
FBS (mmol/L)	9.15 (3.19)	10.32 (5.66)	-1.17 (-3.22, 0.87)	-1.14 (61)	0.259
HbA1c (%)	9.18 (2.05)	9.87 (2.52)	-0.69 (-1.72, 0.33)	-1.35 (78)	0.180
TC (mmol/L)	4.49 (1.37)	4.74 (1.59)	-0.25 (-0.91, 0.41)	-0.74 (78)	0.457
LDL (mmol/L)	2.47 (1.12)	2.66 (1.40)	-0.19 (-0.75, 0.38)	-0.66 (78)	0.508
TG (mmol/L)	1.65 (0.89)	1.63 (1.01)	0.02 (-0.40, 0.44)	0.09 (78)	0.924
Creatinine	90.17 (31.84)	85.93 (25.73)	4.25 (8.64, 17.14)	0.65 (78)	0.513
AST	26.43 (11.31)	26.45 (11.79)	-0.03 (-5.17, 5.12)	-0.01 (78)	0.992
ALT	27.80 (17.64)	30.08 (17.31)	-2.28 (10.05, 5.50)	-0.58 (78)	0.562

*Independent T-test.

**Table 3:** Comparison of Rv-DQoL over 12 weeks between DSPN patients taking a fixed dose combination of ALA and B vitamins *versus* placebo (*n*=80).

**Variables/Visit**	**Mean (SD)**		**Mean diff** **(95%CI) ^a^**	**F-Stat (df)^a^**	***p*-value^a^**
	**Intervention** **(*n*=40)**	**Placebo** **(*n*=40)**			
**Score Rv-DQoL [DQOL within visit: F-Stat (df) =55.16 (2,156); *p* <0.001]**					
Baseline	29.50 (5.25)	29.88 (5.65)	-0.38 (-2.80, 2.05)	1.91 (2,156)	0.151
6 weeks	25.87 (4.28)	26.73 (4.28)	-0.86 (-2.76, 1.04)		
12 weeks	24.37 (3.43)	26.39 (4.02)	-2.02 (-3.68, 0.35)		

**Note:** ^a^Repeated measures ANOVA.

**Table 4:** Comparison of metabolic parameters over 12 weeks between DSPN patients taking a fixed dose combination of ALA and B vitamins *versus* placebo (*n*=80).

**Variables/Visit**	**Mean (SD)**		**Mean Diff** **(95%CI)^a^**	**F-Stat** **(df)^a^**	***p*-value^a^**
	**Intervention** **(*n*=40)**	**Placebo** **(*n*=40)**			
**BMI [BMI within visit: F-Stat (df) =1.13 (2,156); *p*-value =0.325]**					
Baseline	29.21 (3.37)	27.80 (6.04)	1.41 (-0.76, 3.59)	1.28 (2, 156)	0.280
6 weeks	29.17 (3.31)	28.17 (5.48)	1.00 (-1.01, 3.02)		
12 weeks	29.2 (3.128)	28.24 (5.56)	0.97 (-1.03, 2.98)		
**SBP [SBP within visit: F-Stat (df) = 18.08 (2, 156); *p* -value= <0.001]**					
Baseline	138.00 (18.00)	135.43 (20.40)	2.57 (-5.99, 11.14)	1.27 (2, 156)	0.285
6 weeks	130.27 (17.16)	131.77 (17.41)	-1.50 (-9.20, 6.20)		
12 weeks	128.80 (19.15)	125.68 (14.29)	3.12 (-4.40, 10.64		
**DBP [DBP within visit: F-Stat (df) = 4.93 (2, 156); *p*-value =0.008]**					
Baseline	78.45 (11.12)	79.55 (11.45)	-1.10 (-6.13, 3.93)	0.50 (2,156)	0.606
6 weeks	75.06 (11.64)	78.36 (9.82)	-3.30 (-8.09, 1.49)		
12 weeks	74.55 (11.15)	75.82 (10.08)	-1.27 (-6.00, 3.46)		
**FBS (mmol/L) [FBS within visit: F-Stat (df) = 0.38 (1,78); *p*-value =0.536]**					
Baseline	9.15 (3.19)	10.32 (5.66)	-1.17 (-3.22, 0.87)	0.088 (1, 78)	0.768
12 weeks	8.99 (3.51)	9.87 (4.84)	-0.88 (-2.76, 1.00)		
**HbA1C [HbA1C within visit: F-Stat (df) = 0.77 (1, 78); *p*-value = 0.382]**					
Baseline	9.18 (2.05)	9.87 (2.52)	-0.69 (-1.72, 0.33)	0.05 (1, 78)	0.816
12 weeks	9.27 (2.11)	10.04 (2.53)	-0.76 (-1.80, 0.27)		
**TC (mmol/L) [TC within visit: F-Stat (df) = 0.21(1,78); *p*-value = 0.643]**					
Baseline	4.49 (1.37)	4.74 (1.59)	-0.25 (-0.91, 0.41)	0.01 (1, 78)	0.969
12 weeks	4.45 (1.33)	4.69 (1.59)	-0.24 (-0.89, 0.41)		
**LDL-C (mmol/L) [LDL-C within visit: F-Stat (df) =0.18(1,78); *p*-value = 0.672]**					
Baseline	2.47 (1.12)	2.66 (1.40)	-0.19 (-0.75, 0.38)	0.08 (1, 78)	0.774
12 weeks	2.53 (1.25)	2.67 (1.38)	-0.14 (-0.722, 0.45)		
**TG (mmol/L) [TG within visit: F-Stat (df) = 3.13(1,78); *p*-value =0.080]**					
Baseline	1.65 (0.89)	1.63 (1.01)	0.02 (-0.40, 0.44)	0.49 (1, 78)	0.483
12 weeks	1.74 (1.09)	1.83 (1.23)	-0.09(-0.61, 0.42)		

**Note:** ^a^Repeated measures ANOVA.

**Table 5:** Side effects of medication (*n*=80).

**Side Effects**	**Intervention (*n*=40)** ***n* (%)**	**Placebo (*n*=40)** ***n (*%)**
**Visit 2**		
Nausea	6(15.0)	2(5.0)
Itchiness	2(5.0)	1 (2.5)
Dizziness	2(5.0)	1 (2.5)
Light headedness	2(5.0)	1 (2.5)
Lethargy	1 (2.5)	1 (2.5)
Diarrhea	1 (2.5)	
Heat sensation	1 (2.5)	
**Visit 3**		
Nausea	1(2.5)	1(2.5)
Itchiness	0	1(2.5)

## Data Availability

The data of current study are available from corresponding author, [N.M.], on a reasonable request.

## LIST OF ABBREVIATIONS

ALA: Alpha-Lipoic Acid
AST: Aspartate aminotransferase
ALT: Alanine aminotransferase
BMI: Body Mass Index
BP: Blood Pressure
CONSORT: Consolidated Standards of Reporting Trials
DPN: Diabetic Peripheral Neuropathy
DSPN: Distal Symmetric Polyneuropathy
DBP: Diastolic Blood Pressure
FBS: Fasting Blood Sugar
FLP: Fasting Lipid Profile
HbA1c: Glycated Hemoglobin
HDL-C: High-Density Lipoprotein Cholesterol
LDL-C: Low-Density Lipoprotein Cholesterol
LFT: Liver Function Tests
NSS: Neuropathy Symptom Score
NDS: Neuropathy Disability Score
MNSI: Michigan Neuropathy Screening Instrument
RFT: Renal Function Test
SBP: Systolic Blood Pressure
TSS: Total Symptoms Score
T2DM: Type 2 Diabetes Mellitus
TC: Total Cholesterol
TG: Triglycerides
QoL: Quality of Life
Rv-DQoL: Revised Diabetes Quality of Life Questionnaire

## STANDARDS OF REPORTING

CONSORT guidelines were followed.
